# Current Challenges in Translating Tissue-Engineered Heart Valves

**DOI:** 10.1007/s11936-017-0566-y

**Published:** 2017-08-07

**Authors:** O. M. J. A. Stassen, D. E. P. Muylaert, C. V. C. Bouten, J. Hjortnaes

**Affiliations:** 1 Department of Biomedical Engineering, Eindhoven University of Technology, PO Box 513, 5600 MB Eindhoven, The Netherlands; 2 Department of Cardiothoracic Surgery, University Medical Center Utrecht, Utrecht, The Netherlands; 3 Institute for Complex Molecular Systems, Eindhoven University of Technology, Eindhoven, The Netherlands; 4 Regenerative Medicine Center Utrecht, University Medical Center Utrecht, Utrecht, The Netherlands

**Keywords:** Tissue-engineered heart valves, Heart disease, Valvular heart disease

## Abstract

Heart valve disease is a major health burden, treated by either valve repair or valve replacement, depending on the affected valve. Nearly 300,000 valve replacements are performed worldwide per year. Valve replacement is lifesaving, but not without complications. The in situ tissue-engineered heart valve is a promising alternative to current treatments, but the translation of this novel technology to the clinic still faces several challenges. These challenges originate from the variety encountered in the patient population, the conversion of an implant into a living tissue, the highly mechanical nature of the heart valve, the complex homeostatic tissue that has to be reached at the end stage of the regenerating heart valve, and all the biomaterial properties that can be controlled to obtain this tissue. Many of these challenges are multidimensional and multiscalar, and both the macroscopic properties of the complete heart valve and the microscopic properties of the patient’s cells interacting with the materials have to be optimal. Using newly developed in vitro models, or bioreactors, where variables of interest can be controlled tightly and complex mixtures of cell populations similar to those encountered in the regenerating valve can be cultured, it is likely that the challenges can be overcome.

## Introduction

Heart valve disease is a major health burden. The disease encompasses various defects of one or more of the four heart valves that, when in a severe state, can hamper proper blood flow through the heart. In the adult, the mitral valve and the aortic valve are the most commonly affected, with the main defects of the valves being either stenosis (incomplete opening) or regurgitation (incomplete closing). There are multiple causes of heart valve disease, with the most prevalent causes being bacterially induced acute rheumatoid fever (predominantly in lower-income countries) to age-related degeneration of the aortic and mitral valve (predominantly in higher-income countries) [1]. The main current therapeutic options are valve replacement or valve repair, with valve repair being an increasingly preferred therapy for mitral valve regurgitation [2]. Valves can be replaced by a mechanical valve or a bioprosthetic allograft (porcine) valve, or, in rare cases, a homograft (donor) valve. This replacement procedure is performed nearly 300,000 times globally each year [3]. Valve replacements are lifesaving, but not without complications: mechanical valves require lifelong anticoagulation, whereas allografts can undergo calcification or mechanical failure. This can result in a 50% structural valve degeneration rate within 10 years [4]. In addition, both the mechanical replacement, the allograft, and the homograft have no capacity for growth, a major problem in the pediatric population suffering from congenital heart valve disease. To address this issue, the tissue-engineered heart valve (TEHV) has been under investigation for over 30 years as an alternative replacement therapy, starting with in vitro endothelialization of biological valves [5]. Initial trileaflet TEHV consisting of autologous cells seeded in vitro on a preshaped biodegradable scaffold to form a non-immunogenic heart valve graft mimicking the native valve with a capacity to grow maintained functional up to 20 weeks [6]. In the development of TEHV, there has been much attention to the load-bearing function, extracellular matrix (ECM) formation, remodeling, and cellular behavior of TEHVs, all on a macroscopic scale of the tissue. This allowed the TEHVs to be improved for materials used, scaffold design, cells used for seeding, and culturing conditions, to obtain the most robust valve for implantation [7].

As the heart valve is a highly mechanical tissue, exposed to both flow and stretch, important improvements in tissue structural integrity were made when cell culture of the scaffolds was performed under mechanical stimulation, resulting in improved cell and ECM organization leading to better mechanical performance [6]. Although these studies were instrumental in advancing the development and understanding of TEHV technology, these living valves have several practically insurmountable challenges: strict regulations around therapies with living material, the complexity of in vitro culturing, and the logistical problems due to the inability to store valves all make it difficult to commercially implement the TEHV as heart valve therapy. These challenges are currently addressed in two ways, either by treating the TEHV with a decellularization step (dTEHV), or by implanting a scaffold graft directly into the patient for in situ conversion into a living tissue by the host’s cells (in situ TEHV). Decellularization removes the native cells and preserves the ECM generated in an in vitro bioreactor [8]. Cells from the host infiltrate the graft and form a novel autologous living heart valve. Before implantation, the dTEHVs can be stored and therefore are easier to translate to the clinic. The partial degradation of scaffold material and biological functionalization with ECM of the graft in the bioreactor prepares the dTEHV optimally for biocompatibility at implantation. Both in ovine and non-human primate models, these dTEHV have successfully replaced pulmonary heart valves with in vivo functionality of up to 24 weeks [9, 10••]. Still, the logistics of generating dTEHVs is complex and costly due to the bioreactors. To prevent this complexity, the therapy of cell-free, fully synthetic, in situ TEHV is now gaining momentum.

For in situ TEHV, the main advantage is that no in vitro culture is required at all. The lack of biological components on the scaffold reduces the immunogenicity of the in situ TEHV. A main drawback is that the formation of the novel tissue depends on the intrinsic regenerative capacity of the host. Especially in cases of congenital heart valve disease, metabolic disease, or immunologic defects, this may result in disrupted formation of tissue [11]. There has been much progress in defining the production conditions of both dTEHV and in situ TEHV that result in functionality in vivo, but this is a multiscale challenge, wherein mechanistic understanding of the contribution of all the biological, chemical, and mechanical contributors to the regenerating heart valve niche is virtually missing. Therefore, it is imperative to supplement the macroscopic understanding of heart valve mechanics and biological processes with the microscopic understanding of cellular biology, cellular signaling, and mechanics, leading to a homeostatic tissue. Important progress in in situ TEHV was recently published in a study in sheep, that showed stability and functionality of in situ TEHV at 12 months after implantation, without formation of pathological calcifications [12••].

**Fig. 1:**
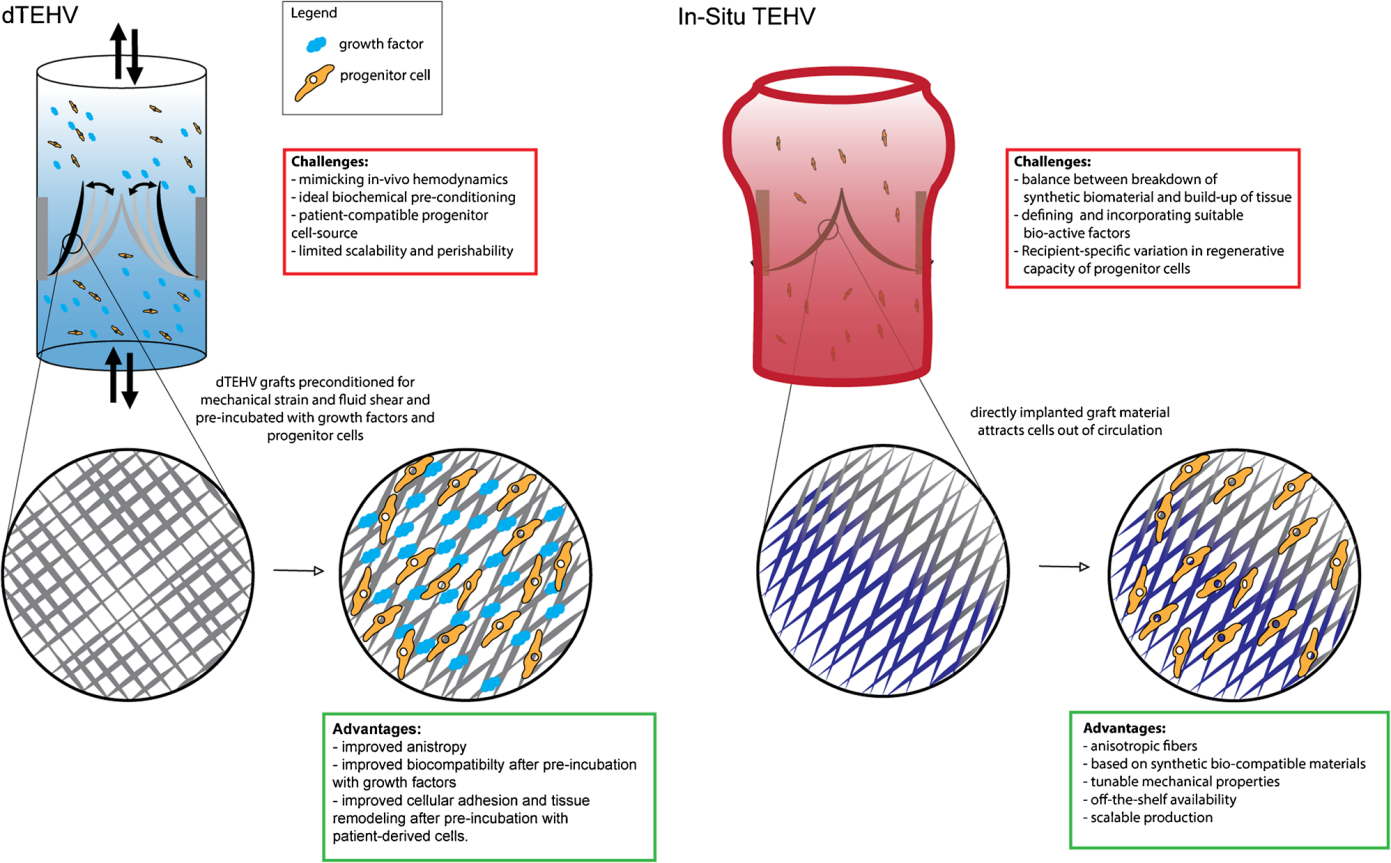
dTEHV and in situ TEHV.

This review aims to address the current challenges of present TEHV strategies, which remain to be overcome in order to achieve successful translation of TEHV to the clinic (Fig.1).

## Challenges of in situ TEHV translation

### Patient-related challenges to tissue engineered heart valves

The concept of in situ tissue engineering depends on both the materials used to build the grafts and the host response to the implanted graft. Considering that the complete cellular contents of a self-seeding graft are derived from the recipient, the quality of these cell sources will directly influence the success rate of the grafts. A major challenge to tissue engineering strategies is to determine which patient-related factors will influence the process of repopulating the cell-free grafts in situ, and how to anticipate these factors in the design of the graft. Though the precise origin and character of the cellular response to scaffold material is difficult to define, it is generally assumed that a population of progenitor cells will be required to achieve the development of a living heart valve replacement. A number of risk factors have been associated with impaired function of progenitor cells in those patient populations that will benefit from cardiovascular tissue engineered grafts. Risk factors such as diabetes mellitus and age are associated with lower levels of circulating progenitor (CD34+) cells [13].These risk factors may have a direct effect on the functionality of the progenitor cells, reducing their migratory or prolific capacity [13, 14]. Progenitor cells derived from patients with type II diabetes adhere less well to endothelial cells and are less capable of participating in the formation of vascular structures in Matrigel assays [15]. In self-seeding grafts, cell migration is paramount to the repopulation of the cell-free materials. Cellular migration is related to the number of CD34+ cells, which are significantly lower in smokers and individuals with a family history of coronary artery disease [14]. Any patient-related factor that will impede progenitor migration is therefore a major challenge to the success of TEHVs, if not accounted for properly.

A patient population that might benefit greatly from living cardiovascular replacement grafts are patients with congenital disease. Encouragingly, there is evidence that children have significantly higher levels of CD34+/KDR+ cells, suggesting a greater capacity for regeneration and possibly a higher likelihood of successfully populating cell-free grafts [16]. In mature patients, there may be a potential for maximizing the number and function of progenitor cells prior to implanting cell-free grafts by administering mobilizing agents. Granulocyte colony stimulating factor has previously been administered to patients to maximize the CD34+ cell response to myocardial infarction. A tripling was found of the maximum white blood cell count with a 5 to 7 fold increase in CD34 cells after a 4 to 5 day treatment schedule. While this increase did not significantly improve left ventricular function in this study, it demonstrates that increasing the number of CD34+ progenitor cells in mature patients is feasible and safe [17]. Screening and potentially pre-treatment of patients prior to implantation of TEHVs, therefore, will likely be an important step in bringing tissue-engineered cardiovascular grafts to the clinic.

### Cell biology-related challenges

The cellular niche is the most important contributor to the tissue formation of a TEHV. A great diversity of circulating cells come into contact with the scaffold after implantation, and the respective contribution of the different cell types to tissue generation is only beginning to be understood. For many different tissue engineering applications, the first reaction after implantation is the infiltration of immune cells initiating a foreign body response. Infiltrating monocytes/macrophages can subsequently assume a phenotype on a spectrum ranging from pro-inflammatory cells to tissue regenerating cells, the M1 to M2 phenotype. In this simplified characterization classical, interferon-γ activated, M1-macrophages represent the inflammatory end of the spectrum, whereas alternatively, interleukin-4 activated, M2-macrophages represent the regenerating end, although current insights reveal the need for characterization by molecularly defined complex activation states [18]. These immune cells will slowly degrade the scaffold material and replace it with tissue. Engineering methods to control this balance to standardize patient outcome is vital. Traditionally, cytokines such as interleukins and IFNγ play an important role in directing macrophage fate [18], but other signaling pathways are also involved. For instance, a recent study in a mice myocardial infarction model revealed that removing a Wnt transporter, Wntless, results in a shift towards M2 macrophages leading to increased angiogenesis in the infarct region [19]. The Notch signaling pathway is also involved in macrophage polarization. Activation of the pathway is associated with M1 polarization and can even overrule other cytokine inducers applied [20, 21]. This has been successfully investigated as a possible therapeutic target in reducing vein graft failure. Targeting Dll4 of either endothelial or macrophage origin, indicated that the Dll4 presented by macrophages contributed to vein graft lesions [22]. This exemplifies that macrophage fate can be controlled in many ways; however, the successful and robust implementation of this control requires clever biological engineering and thorough testing.

Early tissue formation ideally transitions into stable tissue, leading to a homeostatic valve that mimics the native, healthy valve as closely as possible. In the native valve, the main cell types are the valvular endothelial cells (VECs) and the valvular interstitial cells (VICs). The interaction between VECs and VICs maintains the structural integrity of the native heart valve. Both VECs and VICs come in a multitude of different phenotypic variants, with five VIC subtypes described with various functions in development, physiology and pathology [23, 24]. In vivo experiments with dTEHV show that at stages of 4 weeks valves are already partially lined with endothelium and the interstitium contains vimentin positive, αSMA negative cells, possibly indicative of quiescent fibroblast-like cells, although αSMA positive cells are also seen indicating the presence of activated contractile cells [9, 10••]. Specific characterization of the specific cell type in the interstitium is challenging. How the transition from early tissue formation to a mature valve takes place is poorly understood, but may be initiated by endothelialization from circulating endothelial progenitor cells, followed by endothelial to mesenchymal transition (EndoMT). The process of EndoMT also plays an important role in both early and late stages of native valve development [25, 26]. If early stages of tissue formation can be controlled, it is possible that the subsequent maturation of the in situ TEHV introduces less variation across patients; however, experiments to test this are complex. As the scaffold is already partially degraded at these stages, this step in the tissue-forming process is more difficult to control from the scaffold design and may require pharmacological interventions that in their turn are more difficult to specifically deliver to the heart valve niche.

### Challenges in cellular biomechanics

Heart valves are tissues that are highly exposed to mechanical cues. During each cardiac cycle, the valves ensure the correct direction of the blood flow through the heart’s atria and ventricles. Alternating systole and diastole creates pressure gradients across the valves. When this gradient is against the correct flow direction, the valves close and are stretched, stopping the blood flow. When the pressure gradient is in the correct flow direction, the valves open, blood flows through the orifice and stretch on the leaflets is released. After implantation of an in situ TEHV, the scaffold material is the main load-bearing structure. As the material is degraded and replaced by ECM and cells, the mechanical properties of the valve change, but integrity has to be maintained throughout the whole process. As the ECM becomes the load-bearing component of the valve, these loads have continuous effect on the organization of cells and the remodeling of the ECM, similar to the native heart valve [27]. This has large effects on the mechanical cues experienced by the cells in these valves. To mimic these conditions and to precondition TEHV and dTEHV for implantation, in vitro bioreactors are implemented that mechanically load the tissue. In vivo experiments, however, resulted in regurgitation and leaflet retraction [28, 29]. Leaflet retraction in TEHV and dTEHV is an effect of the imbalance of the load exerted on the valve under diastole and the contractility and anisotropy of the cells in the leaflets [30]. Approaches to understand these effects through computational modeling revealed fundamental improvements of design to minimize regurgitation through balancing of hemodynamic forces and tissue organization. Subsequent redesigning of valve geometry and tissue anisotropy have led to improved valve coaptation area and tissue stability and may highlight the way forward in obtaining ideal valve design [31, 32•]. How the balance of macroscopic and microscopic forces affects in situ TEHV remains to be investigated.

Several studies investigated the cellular mechanisms pathways in cells responding to mechanical forces as predictors of cell fate, as mechanical cues can contribute to pathological states of the cells [33, 34].

The Notch signaling pathway, mentioned before in macrophage polarization, is emerging now as a possible mediator of mechanotransduction. The Notch signaling pathway is a direct cell-cell contact signaling pathway of great importance in general organization of tissues, especially cardiovascular development. Notch is known to be crucial in various stages of heart valve biology, initially in the formation of endocardial cushions and control of EndoMT, up to the maintenance of cells in non-calcific state in the mature valve [26]. Defects in the Notch signaling pathway, both in the receptors (Notch 1–4), the ligands (Jag1 and 2, Dll1, 3 and 4), as well as the effector genes (Rbpjk) result in congenital defects including outflow tract malformations and heart valve defects [35]. Notch1 was the first gene found to cause familial BAV and calcific AoVD [36]. Defects in Notch signaling alter cellular response in both to VICs under strain and VECs under shear forces, predisposing cells for a calcific fate [37, 38]. This is in line with other tissues where the Notch signaling pathway is directly responsive to mechanical cues, although the functional outcome varies across tissues, indicating complex regulatory mechanisms [39, 40]. Altered cellular responses to mechanical cues in tissue-forming cells with Notch defects make this another example of congenital defects that may complicate the implementation of in situ TEHV.

### Guiding tissue formation by bioactive materials

Another way to guide the tissue formation, in addition to preconditioning the patient to mobilize the proper cell populations, is by modifying material to be optimally suited to guide tissue formation through cell fate choices. The main body of research into bioactivating polymeric materials has focused on reproducing extracellular matrix elements such as collagens [41] and glycosaminoglycans [42] capable of adhering cells [43]. This approach is based on the concept that tissues derive their mechanical and biological characteristics from a cellular population on one hand, and a structural matrix to adhere to on the other. In in situ tissue engineering, however, a third element, namely that of novel tissue development, plays a major role. A very high level of tissue development is needed to produce enough ECM to fully take over from polymeric graft material and stimulate and maintain a cellular population. The requirements of in situ tissue engineered grafts have a lot in common with wound healing and tissue repair. Choosing candidates for bioactivity in cell-free constructs, therefore, should be based not only on cellular adhesion but also focus on triggering the developmental cues required to build up a fully functional biological tissue from scratch. Inspiration for biologically active molecules that may help orchestrate an appropriate tissue development may be found in repair processes such as ischaemic damage, which is known to trigger the mobilization and homing of cell populations capable of regenerating tissue and repairing damage [44]. Chemokines are molecules that are capable of both inducing cellular migration and cellular development. Proteins such as stromal cell-derived factor 1a (SDF1a) and monocyte chemoattrative protein1 (MCP1) have previously been investigated in an in situ tissue engineering context [45••, 46]. Targeting Notch is notoriously difficult due to aspecificity following from the multitude of receptors and ligands involved and the risk of off-target effects, but novel engineered peptides may hold potential for harnessing these developmental signals [47•, 48]. In addition, factors such as platelet-derived growth factor PDGF [49] and vascular endothelial growth factor (VEGF) [50] show promising results in incorporating specific bioactivity into synthetic materials using a wide range of delivery methods [44]. An exciting though challenging aspect of in situ tissue engineering is the search for molecules that both attract and orchestrate populations of cells to go through the developmental stages of building up a previously non-existent living tissue.

### Scaffold-related challenges

When moving from in vitro engineered dTEHV to in situ TEHV, the challenges for the scaffolds used become apparent. Importantly, the biological processes related to valvular tissue formation and regeneration need to be stimulated, orchestrated, and controlled with a single, non-living and preferably degrading scaffold. From a materials point of view, these challenges are multidimensional. At the microscopic level, it is necessary to build the right niche for spatiotemporal control of cell recruitment, behavior (proliferation, differentiation, matrix production), quiescence, maintenance, and growth. This can be done via manipulating the biological, biochemical, and biophysical properties of scaffolds in close interplay with physiological environments [51]. The final purpose being to guide the tissue towards the functional layers found in the native heart valve, the ventricularis, fibrosa, and spongiosa, and the corresponding cell/ECM composition of these layers.

On the mesoscale level, it is important to control scaffold mechanical behavior. Opening and closing of the valve induce local deformations, and the stress distribution across the valve and subsequent anisotropy can make the difference between a regurgitating or a tightly sealed valve. The scaffolds degradation rate is of importance to maintain integrity in early stages of tissue formation and is mainly dependent on materials and processing: e.g., electrospinning, printing, or molding.

At the macroscale level, the handling and implantation of the scaffold are relevant during surgery, either as a surgical implant or minimally invasive delivery, e.g., as transcatheter valve replacement [52]. In the case of minimally invasive delivery, after implantation, the valve has to deploy from the shape of delivery into its functional shape. These properties are difficult to control with decellularised matrices, but synthetic matrices can offer full control at each length scale and with extraordinary reproducibility.

### Challenges in modeling tissue regeneration

To accelerate the process of material design across length scales, bioengineering approaches like high-throughput analysis are crucial. In vivo experiments need to be the last step before translation occurs. But much information on the posed challenges can be obtained in vitro. Particularly, when looking at tissue regeneration, in vitro studies are important. Valvular tissue regeneration can be defined as the mechanism of maintaining valvular structural integrity by valvular cells. The process of activating and de-activating VICs, responsible for remodeling the valve ECM, is key to maintaining tissue homeostasis, and thus, also a vital challenge to understand when engineering a heart valve.

In vitro models allow for more accurate isolation and manipulation of independent variables controlling tissue formation, however, not in its traditional form. In vitro systems have typically used Petri dishes to study the role of the valvular cell populations and its role in tissue formation. However, the intrinsic unnatural environment of stiff substrates, and the two-dimensional environment that cultured cells reside in limits this approach. As described earlier, VICs harbor a great degree of mechanosensitivity, leading to uncontrollable phenotypic changes in two-dimensional culture systems [53]. As such, the field has looked at developing three-dimensional in vitro systems able to not only independently modulate the VIC and VEC phenotype, but also simulate the entire cellular driven process of valve homeostasis. Such a three-dimensional in vitro approach will allow to study the native valve tissue formation outside of the human body, ultimately providing us with a much needed map of guiding human tissue formation.

To overcome this challenge, hydrogel micro-engineering has emerged. Hydrogels can be designed using natural proteins, such as collagen, hyaluronic acid, and elastin, to recapitulate vital environmental cues in native tissues [54–56]. Hydrogels have yielded a great deal of interest due to their ability to be chemically and mechanically tailored to specific needs, and in this case, understanding valvular tissue formation.

Mounting evidence has shown that mechanical properties of the hydrogel can activate and modify intracellular pathways and alter VIC function. To this end, hydrogel studies have shown to maintain a quiescent VIC culture in three-dimensional cultures, similar to a healthy native valve [55, 56]. Controlling the mechanics and substrate stiffness in these three-dimensional models identified the PI3/AKT pathway as an elasticity sensitive pathway important for preservation of native VIC phenotype [57]. Conversely, changing hydrogel substrate and mechanics, mechanisms of myofibroblast activation of VICs have been elucidated. Shear stress [58, 59], changes in substrate stiffness [60], cellular proximity, paracrine regulation of VICs by VECs [61••, 62], and ECM disruption [63], all contribute to the activation of VICs.

Not only understanding what leads to ECM deposition by VICs in native tissue, is key to understand neo-tissue formation in a TEHV, but also what is needed to maintain a healthy tissue homeostasis, and thus preventing activated cells to keep depositing ECM, reminiscent of fibrosis. Furthermore, most three-dimensional culture systems utilize a single natural protein, such as collagen or hyaluronic acid, and work remains to be done to combine all native natural valve proteins into one three-dimensional culture system reminiscent of the heart valve ECM. In addition, challenges remain to simulate the valvular hemodynamic environment in a three-dimensional culture system. To this end, organ-on-a-chip technology and different pulsatile bioreactors are being developed.

## Conclusion

TEHV differs vastly from the current standard of mechanical implants or allografts, bringing new perspectives and challenges to overcome (Table 1). Challenges arise from the complexity of the regeneration process. Patient variation and comorbidity affecting regeneration, the multiple phases in tissue formation, and the limited control that can be exerted on the regeneration process by incorporating guiding cues in the scaffold are the main challenges to be overcome. Considering the current treatments are lifesaving, the strongest challenge perhaps is that the TEHV will have to perform at least as good as the current treatments, with less complications such as the use of anticoagulation or reoperation. Translation of TEHV of all types to the clinic will remain a process with uncertainties, due to the large differences between the large animal models and the patient. Using models where variables of interest can be controlled tightly, and wherein complex mixtures of cell populations similar to those encountered in the regenerating valve can be cultured, we expect that all challenges can be overcome and the TEHV will become a novel lifesaving therapy.

**Table 1:** Identified challenges in the translation of (in situ) tissue-engineered heart valves

Origin of challenge	Nature of challenge
Patient	Variations in regenerative capacity make outcomes of valve engineering unpredictable
Patient	Finding ways to mobilize the proper cell population to kickstart tissue regeneration
Early tissue formation	Valve must stay intact throughout scaffold degradation/tissue formation
Late tissue formation	Reaching a steady state of tissue growth, apoptosis, remodeling, and quiescence
Transition of early to late tissue formation	Guiding the process of early formation to such an extent that healthy late tissue formation builds upon the early tissue in all patients
Material	Identifying and controlling the biomechanical cues that can guide cell fate based on material mechanical or topological properties
Material	Choosing the bioactive compounds that benefit the maximum amount of patients by standardizing the tissue generating process by attracting cells and guiding cell fate
Material	Finding the material that has all the required properties: optimal robustness in handling and implantation, correct degradation rate, while allowing incorporation of topological, biomechanical and biochemical cues
In vitro models	Controlling the relevant variables for understanding and ultimately predicting the impact of TEHV designs and therapies on outcomes
